# Identification of DMP1 as Novel p53 Repressed Transcriptional Target

**DOI:** 10.3390/ijms27031344

**Published:** 2026-01-29

**Authors:** Jun Xu, Christian Britschgi, Gustav Arvidsson, Deborah Krauer, Inti Zlobec, Bruce E. Torbett, Mario P. Tschan

**Affiliations:** 1 Institute of Tissue Medicine and Pathology, Division of Experimental Pathology, University of Bern, CH-3008 Bern, Switzerland; jun.xu@unibe.ch (J.X.);; 2 Graduate School for Cellular and Biomedical Sciences, University of Bern, CH-3012 Bern, Switzerland; 3 Department of Medical Oncology, Inselspital, Bern University Hospital, University of Bern, CH-3010 Bern, Switzerland; 4 Department of Immunology and Microbiology, The Scripps Research Institute, La Jolla, CA 92037, USA; 5 Department of Pediatrics, School of Medicine, University of Washington, Seattle, WA 98195, USA; 6 Center for Immunity and Immunotherapies, Seattle Children’s Research Institute, Seattle, WA 98101, USA

**Keywords:** DMP1, DMTF1, hDMP1, mDmp1, negative feedback, p53

## Abstract

The transcription factor *DMP1* is a positive regulator of the tumor suppressor ARF, which in turn controls cell-cycle progression and/or apoptosis through p53. Given the role of DMP1 in the ARF-p53 pathway, we investigated whether the p53 transcription factor might regulate DMP1 expression. We found that endogenous human *(h)DMP1* mRNA levels were significantly decreased upon induction of the temperature-sensitive p53^Val135^ in normal fibroblasts. Consistent with this observation, a p53 knockdown in MCF7 breast cancer cells resulted in increased *hDMP1* mRNA and protein levels. At the molecular level, we found that p53 directly repressed the hDMP1 promoter activity by up to 90%. This repression was not mediated by p53 binding to the two putative p53-binding sites in the hDMP1 promoter. Instead, deletion analysis revealed a 300bp region containing an EGR1/SP1 binding site that is required for p53-dependent inhibition of hDMP1 promoter activity. Using Sp1-deficient SL2 insect cells, we confirmed that p53-mediated repression of hDMP1 is dependent on Sp1. Furthermore, chromatin immunoprecipitation demonstrated SP1 binding to the hDMP1 promoter. Together, our findings identify an Sp1-dependent, p53-mediated repression of DMP1.

## 1. Introduction

P53 is a well-known tumor suppressor that plays a key role in the prevention of tumor formation. The p53 transcription factor is activated upon cellular stress and induces growth arrest or apoptosis depending on the severity of the damage and the cell context. P53 functions are mainly mediated through transcriptional regulation of its downstream target genes. For example, p53 transactivates molecular pathways important for cell cycle arrest, such as p21 and 14-3-3s, or for induction of apoptosis, such as Bax, PUMA, NOXA and Fas. However, p53 also transcriptionally represses survival genes such as Bcl-2 and survivin [1,2].

The cyclin D-binding myb-like protein 1 (designated *mDmp1* for mouse and *hDMP1* for human; also called *Dmtf1* or *DMTF1*) transcription factor is a critical regulator of cell cycle control and differentiation [3,4] with multiple isoforms [5]. Presently, two target genes of *mDmp1*—the tumor suppressor *Arf* and human *CD13/Aminopeptidase N*—are described and functionally validated [6,7]. Moreover, *mDmp1* is needed for *Arf* activation in response to oncogenic Ras [8]. *Dmp1*-null mice developed spontaneous tumors during their second year of life and showed dramatically accelerated lymphomagenesis in the presences of an oncogenic Eµ-Myc transgene [9,10]. Surprisingly, tumors arose with similar latencies in either *DMP1* hemizygous or null mice without any alterations of the remaining wild-type protein. This indicates that *DMP1* is haplo-insufficient for tumor suppression, that it phenocopies *Arf* function in tumor suppression, and that *DMP1* expression levels play an important role in predisposition to tumorigenesis [11]. This is further supported by a previous publication showing that *mDmp1* is a bona fide tumor suppressor in lung cancer, and that the loss of heterozygosity (LOH) of the *mDmp1* gene is mutually exclusive with LOH of *Arf* or *p53* [12].

Regarding transcriptional regulation of *DMP1* the following mechanisms have been found: (a) induction by oncogenic Ha-Ras^V12^ via jun proteins [8], (b) inhibition by *E2F* transcription factors paralleled by increased proliferation [13], (c) inhibition of *mDmp1* expression by anthracyclin treatment via NF-kB binding to the promoter [14], (d) repression of the human *hDMP1* promoter by the *Wilms’ tumor 1 gene (WT1)* via an EGR/SP1 site [15], (e) activation by HER2/neu through the PI3K-Akt-NF-κB pathway [16], (f) inhibition of hDMP1 promoter by miR-155, miR-675-3p and MiR-6838-5p [17,18,19], and repression of the DMP1 promoter by CD74 [20]. At the protein level, DMP1 binds to the C-terminus of p53, antagonizing MDM2-mediated polyubiquitination and degradation of p53, and promoting its nuclear localization [21]. This interaction also stabilizes the p53–DNA complexes on target promoters [22]. Given the important role of DMP1 in the ARF-p53 pathway, we investigated whether there is a feedback mechanism—either positive or negative—of p53 to DMP1 as shown for other p53 activators [23,24,25].

## 2. Results and Discussion

### 2.1. Expression of p53 Represses Endogenous DMP1 mRNA and Protein

**Figure 1:**
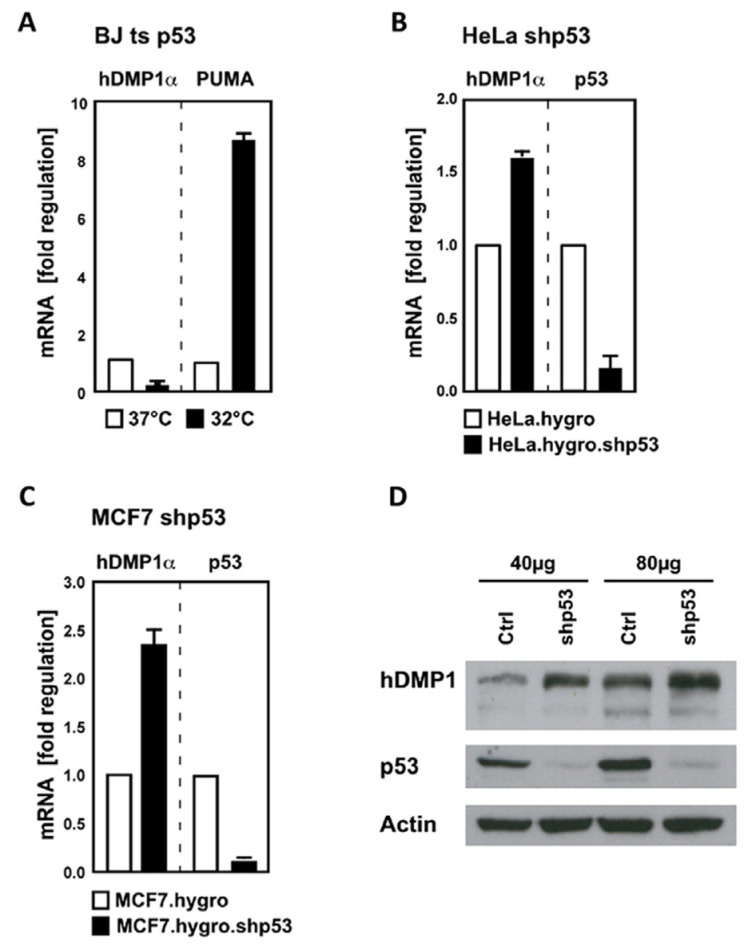
p53 represses hDMP1 expression. (**A**) Full length *hDMP1* and *PUMA* quantitative RT-PCR analysis of mRNA isolated from normal human BJ fibroblasts expressing a temperature-sensitive p53 mutant (p53^Val135^) gene and cultured for 24 h at 37 °C (nonpermissive) or at 32 °C (permissive). Messenger RNA expression levels of *hDMP1* and *PUMA* at 32 °C are indicated as n-fold upregulation normalized to values at 37 °C and to *HMBS* expression. Experiments were performed in duplicates; error bars represent standard deviations. (**B**,**C**) Quantitative *hDMP1* and p53 RT-PCR of RNA isolated from HeLa and MCF7 control (HeLa.hygro, MCF7.hygro) or p53 knockdown (HeLa.hygro.shp53, MCF7.hygro.shp53) cells. Messenger RNA expression levels of *hDMP1* and p53 are indicated as n-fold upregulation compared to control cells. (**D**) DMP1 and p53 protein expression in MCF7 control (MCF7.hygro) or p53 knockdown (MCF7.hygro.shp53) cells. β-actin was used as a loading control.

To interrogate whether p53 regulates *DMP1*, we utilized different p53 expression and knock-down systems. In the first experiment, we transiently transfected p53-null human H1299 non-small cell lung cancer cells with a p53 expression plasmid and found a 72% reduction in endogenous *hDMP1* mRNA levels as compared to mock-transfected cells. To confirm *hDMP1* inhibition by p53 in normal fibroblasts, we utilized human BJ fibroblasts expressing a temperature-sensitive p53^Val135^ mutant. At the permissive temperature, active p53 inhibited endogenous *hDMP1* mRNA up to 73% as measured by quantitative RT-PCR (Figure 1A). As a control for p53 activation, we measured the induction of the known p53 target *PUMA*. Since the previously described experiments relied on ectopic expression of p53, we next evaluated whether decreasing p53 in HeLa cervical and MCF1 breast cancer cells would allow for higher *hDMP1* mRNA expression. Indeed, we found that *hDMP1* transcript levels were 1.6- and 2.4-fold higher in HeLa and MCF7 p53 knockdown cells as compared to control cells, respectively (Figure 1B,C). Moreover, increased *hDMP1* mRNA levels in MCF7 p53 knock-down cells were paralleled by enhanced hDMP1 protein expression (Figure 1D).

Taken together, our findings in both cell lines demonstrated that endogenous *DMP1* levels are repressed in p53 wild-type cells and that inactivating p53 allows for higher *DMP1* mRNA and protein expression.

### 2.2. p53 Represses DMP1 Promoter Acitivity

**Figure 2:**
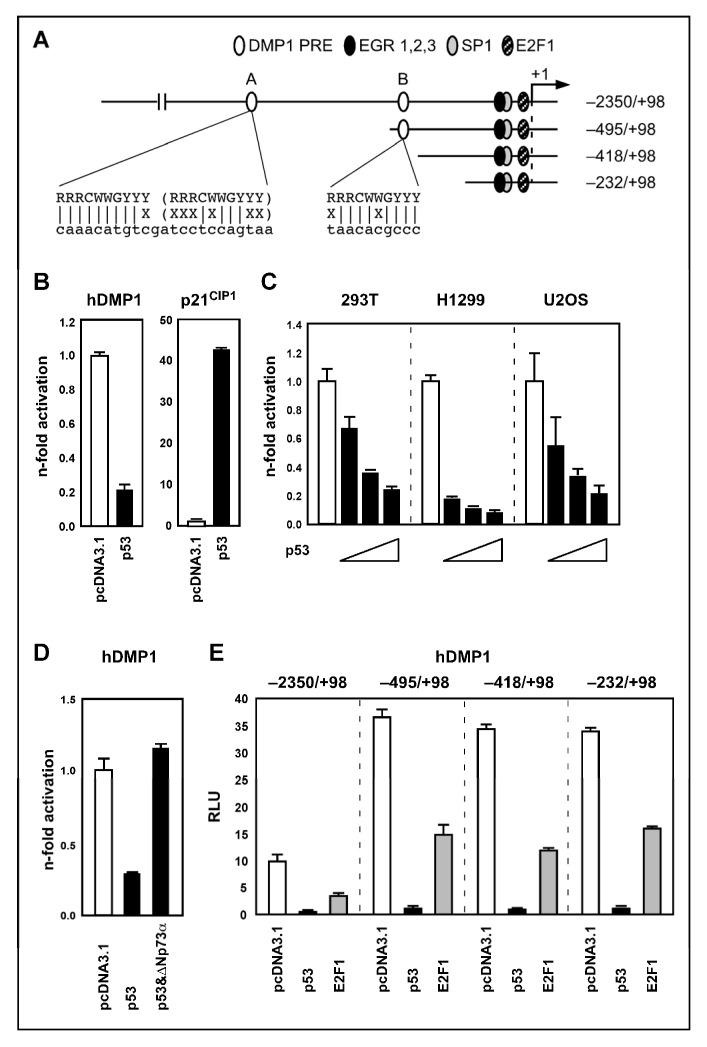
p53 transcriptional repression of the human DMP1 promoter. (**A**) Schematic representation of the human DMP1 promoter structure. Two putative p53 (DMP1 PRE; A and B), EGR1, Sp1 and E2F1 binding sites as well as the transcriptional start site (+1) according to the mouse Dmp1 promoter [8] are shown. Position of progressively truncated hDMP1 promoter constructs are indicated. (**B**) Human embryonic kidney 293T cells were co-transfected with 100 ng hDMP1 or p21^Cip1^ promoter reporter, with 100 ng p53 expression plasmid or empty vector and 10 ng pRL-TK plasmid. Luciferase activity was determined 24 h after transfection. Results were normalized for Renilla luciferase activity, and the fold activation was obtained by setting the value of control as 1.0. All transfection assays were carried out in triplicate. Data are shown as mean ± s.d. (**C**) Dose-dependent repression of hDMP1 promoter activity by p53; 293T human embryonic kidney cells, p53 function inhibited by large T antigen, H1299 non-small cell lung cancer cells, p53-null and U2OS osteosarcoma cells, wild-type p53, were cotransfected with increasing amounts of the wild-type p53 expression plasmid (30, 100, or 300 ng) and the pGL2-hDMP1 reporter plasmid (100 ng). Analysis as described in (**B**). (**D**) Cotransfection of p53 (100 ng) with the dominant negative ∆Np73α (100 ng) in 293T cells abrogated p53 repression on the hDMP1 promoter. Analysis as in (**B**). (**E**) 293T cells were co-transfected with 100 ng of hDMP1 deletion mutants and 100 ng p53 or E2F1 expression plasmids and 10 ng pRL-TK plasmid. Analysis as in (**B**). Results were normalized to Renilla luciferase activity and results are given as relative luciferase activity (RLU).

To determine whether p53 directly repressed the hDMP1 promoter, we inserted a genomic DNA fragment containing 2350 bp in the promoter region and 98 bp downstream of the transcriptional initiation site into the pGL2.basic luciferase reporter vector (Figure 2A; −2350/+98). The promoter activity of this construct was markedly inhibited by p53 when co-expressed in 293T human embryonic kidney cells (Figure 2B). The p21^Cip1^ promoter reporter plasmid was used as positive control for p53 wild-type functionality. Moreover, repression of the hDMP1 promoter reporter was dose-dependent, which was seen in human p53-negative, -inhibited and -positive cells, H1299, a non-small cell lung cancer line, 293T, an embryonic kidney cell line, and U2OS—an osteosarcoma line—respectively (Figure 2C). As expected, the promoterless luciferase pGL2-basic vector showed no promoter activity and could not be repressed by p53. In addition, p53 repression of hDMP1 was reversed by cotransfecting ∆Np73α, a known dominant negative regulator of p53 (Figure 2D).

Since p53 usually transactivates its target genes through direct binding to p53 consensus binding sites [26], we used MatInspector Version 05 [27] and TESS V1.0 software to search for putative p53 binding sites in the *hDMP1* promoter. We identified two sequence motifs in the 2.3 kb *hDMP1* promoter fragment that showed significant homology to the p53 consensus binding site (Figure 2A). However, in vitro gel retardation assays revealed that p53 does not bind to any of these sites (Supplementary Figure S1). These results indicate that hDMP1 repression by p53 did not occur through direct binding of p53 to the promoter.

To localize the p53 responsive element in the *hDMP1* promoter, we next generated a series of *hDMP1* promoter deletion constructs that were composed of various lengths of 5′-promoter sequences fused to the luciferase gene (Figure 2A; −495/+98, −418/+98, −232/+98). The promoter activities of the various deletion constructs were markedly inhibited by p53 co-expression (Figure 2E). Furthermore, the (−232/+98) promoter fragment was sufficient to mediate p53 repression activity. E2F1 co-transfection with the human DMP1 deletion constructs served as control, since E2F1 has been shown to inhibit *mDmp1* promoter activity [13]. Moreover, the shortest construct (−33/+98) lacking an EGR-1/Sp1 site almost completely abolished promoter activity. This is consistent with earlier findings showing the importance of the EGR-1/SP1 site for basal activity of the mouse *Dmp1* promoter [8].

### 2.3. Sp1-Dependent, p53-Induced Inhibition of hDMP1 Promoter Activity

**Figure 3:**
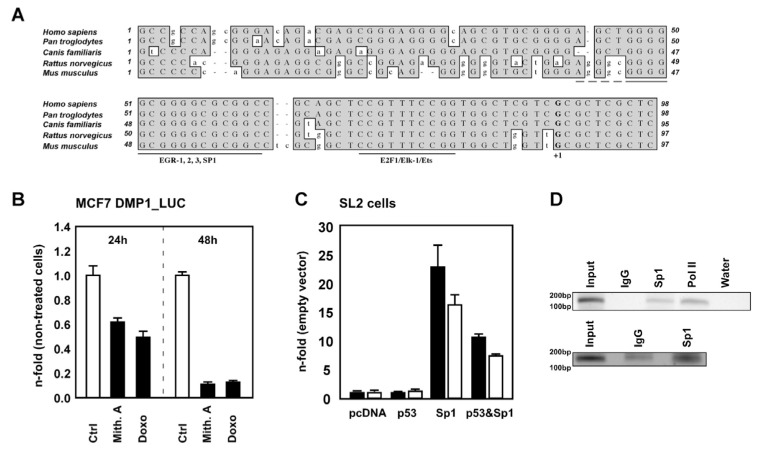
p53 repression of DMP1 is mediated via Sp1. (**A**) Alignment of highly conserved EGR1/SP1 and E2F1 binding sites in the DMTF1 core promoter of human, chimpanzee, dog, rat, and mouse. The bold G indicates the transcriptional initiation site (+1) as determined in the mouse. (**B**) hDMP1 promoter activity is repressed by mithramycin A. MCF7 DMP1_LUC cell were then treated with mithramycin A (200 ng/mL) or doxorubicin (1 µM). After 24 and 48 h cells were harvested and Luciferase activity was measured. Results are shown relative to non-treated controls as mean ± s.d. of three experiments. (**C**) Sp1-dependent p53-induced repression of the (−232/+98) hDMP1 promoter in SL2 *Drosophila* cells. SL2 cells were transfected with 1.2 µg p53, 1.2 µg Sp1 expression plasmids, 2.0 µg hDMP1 promoter deletion construct and 250 ng pRL-TK per six well as indicated. Results are given as n-fold compared to the empty vector transfected control ± s.d. Two independent experiments, represented as black and white bars, of triplicate transfections are shown. (**D**) Chromatin immunoprecipitation assay using 293T cells transiently transfected with Sp1 expression plasmids and antisera against Sp1. Where indicated, Anti-Pol II and IgG served as positive and negative controls, respectively (n = 2).

How might p53 inhibit *hDMP1* promoter activity? Since the EGR-1/SP1 site in the *hDMP1* promoter is essential for its activity, we next tested whether p53-mediated DMP1 repression occurs via Sp1. An important role for the EGR-1/Sp1 regulatory site is further underlined by its high conservation among different species (Figure 3A).

In a first attempt to investigate if the Sp1 class of transcription factors play a role in *hDMP1* gene transactivation, we treated MCF7 DMP1_LUC cells stably expressing the (−495/+98) *hDMP1* promoter fragment fused to the luciferase gene with mithramycin A. Mithramycin A is a drug that binds to GC-rich regions of DNA and blocks Sp1 binding [28]. Thus, if *hDMP1* is a Sp1-transcriptional target, blocking endogenous Sp1 binding to the *hDMP1* promoter will reduce luciferase transactivation. Indeed, MCF7 DMP1_LUC cells showed significant reduction in luciferase activity upon Sp1 inhibition by mithramycin A treatment (Figure 3B). Doxorubicin was used as control, since it was shown that anthracyclin treatment inhibits mDmp1 promoter activity via NF-kB [14].

A necessary role for Sp1 in activating the *hDMP1* promoter via the conserved EGR1/SP1 site was confirmed by use of Sp1-null *Drosophila* SL2 cells for functional analysis of the *hDMP1* promoter. Since SL2 cells lack Sp1 there was no activation of the −232/+98 *hDMP1* promoter reporter. In contrast, cotransfection with Sp1 plasmids restored the Sp1-mediated response. Consistent with the role of p53 in regulating DMP1, co-expression of p53 with the restored Sp1 in SL2 cells resulted in a potent suppressive effect on *hDMP1* promoter activity (Figure 3C). Chromatin immunoprecipitation (ChIP) assays were also undertaken to determine whether Sp1 binds to the *DMP1* promoter in vivo. Nuclear lysates from Sp1 transfected 293T were submitted to Sp1 pull-downs utilizing antibodies against Sp1. The precipitated chromatin was used to amplify a 149 bp fragment of the proximal *hDMP1* promoter containing the EGR-1/SP1 site. As seen in Figure 3D, enrichment of Sp1 at the proximal *hDMP1* promoter relative to the IgG control was seen, thus demonstrating that Sp1 binds to the *hDMP1* proximal promoter.

In summary, we show that p53 expression represses *DMP1* mRNA and protein levels and that reduced p53 levels allow for higher *DMP1* expression in cell lines. Our findings therefore indicate that *DMP1* is subject to p53 regulation and is a newly identified p53-repressed target gene. Consistent with our findings, Yoon et al. [29] observed higher *mDmp1* levels in p53-null mice compared to wild-type mice upon benzene-induced DNA damage. Moreover, we found that p53 represses the DMP1 promoter most likely by interacting with Sp1. This p53-mediated molecular mechanism has been shown to be operative for several p53 repressed genes such as survivin, telomerase, cyclin B1, and protein kinase C α [1,30,31,32,33]. Nevertheless, we cannot exclude the involvement of other transcription factors, for example, AP-1 or Ets-1, that may contribute to p53 repression [34,35].

Notably, we did not assess the effects of mutant p53 on endogenous hDMP1 expression. As repression of DMP1 does not require direct p53 binding to p53 response elements within the promoter region, and given that the mutant p53 has been shown to interact with Sp1, it is conceivable that loss-of-function p53 mutants that lack DNA-binding capacity may still influence DMP1 repression in a tumor context fashion where downregulation of DMP1 is advantageous. The impact of distinct p53 mutations on DMP1 regulation warrants further investigation.

What could be the biological necessity of p53-mediated repression of *DMP1*? At first glance it appears somewhat paradoxical that p53 represses yet another tumor suppressor. On the other hand, it is essential that the potent cell cycle arrest and cell death inducer programs regulated by p53 are under tightly controlled regulation in healthy cells, thereby avoiding the severe consequences of the untimely activation of p53 targets. Our findings are consistent with a growing number of reports suggesting that p53 imparts a negative feedback control of ARF through various pathways [36,37]. We propose that our findings of p53-mediated repression of *DMP1* identifies an additional mechanism whereby control of normal cellular growth is fine-tuned.

## 3. Materials and Methods

### 3.1. Cell Culture

H1299 non-small cell lung cancer, 293T human embryonic kidney, U2OS osteosarcoma, and MCF7 breast cancer cell lines were purchased from the Deutsche Sammlung von Mikroorganismen und Zellkulturen GmbH (DSMZ, Braunschweig, Germany). The p53 status of the cell lines used was as follows: H1299 non-small cell lung cancer cells are p53-null, 293T human embryonic kidney cells express SV40 large T antigen resulting in functional inactivation of p53, U2OS osteosarcoma, and MCF7 breast cancer cells express wild-type p53. The generation of normal human BJ fibroblasts expressing temperature sensitive p53^Val135^ and MCF7 p53 knockdown cells has been described in detail elsewhere [38]. Cells were maintained in Roswell Park Memorial Institute 1640 Medium (RPMI-1640) or Dulbecco’s modified Eagle’s medium (DMEM) supplemented with 10% fetal calf serum (FCS), 50 U/mL penicillin, and 50 µg/mL streptomycin in a 5% or 10% CO_2_-95% air humified atmosphere at 37 °C. Early passage Schneider (SL2) cells were kindly provided by D. Kojic (Institute of Cell Biology, University of Bern) and maintained in Drosophila’s Schneider Insect Medium (Sigma-Aldrich, Buchs, Switzerland) supplemented with 10% FCS, 50 U/mL penicillin, and 50 µg/mL streptomycin without any gas exchange at 25 °C.

### 3.2. Quantitative Real-Time Reverse Transcription-PCR

Total RNA was extracted using the RNeasy Mini Kit and the RNase-Free DNase Set according to the manufacturer’s protocol (Qiagen, Hombrechtikon, Switzerland). Total RNA was reverse transcribed using random primers (Roche Diagnostics, Rotkreuz, Switzerland) and M-MLV reverse transcriptase (Promega, Madison, WI, USA). PCR and fluorescence detection were performed using the ABI PRISM^®^ 7700 Sequence Detection System (Applied Biosystems, Rotkreuz, Switzerland). Primers and probes for full-length *hDMP1* and *HMBS* have been described [15]. For quantification of *BBC3/PUMA* a Taqman Gene Expression Assay was used (Applied Biosystems, Hs00248075_m1). p53 was measured using the UniPrimer detection system [39] and the following primers: Forward 5′-actgaacctgaccgtacaGCGTGAGCGCTTCGAGAT-3′ and reverse 5′-CAGCCTGGGCATCCTTGA-3′. Target gene mean Ct-values were normalized to the respective housekeeping gene (*HMBS*), and then to the experimental control. Obtained values were exponentiated 2^−ΔΔCt^ to be expressed as n-fold changes in regulation compared to the experimental control.

### 3.3. Immunoblot Analyses

Whole cell extracts were prepared using RIPA lysis buffer supplemented with 8M Urea. Blots were incubated with the primary antibodies in TBS 0.05% Tween-20/5% milk overnight at 4 °C, incubated with secondary HRP-coupled anti-rabbit antibody at 1:5000 for 1 h at room temperature, and analyzed chemiluminescently using the ECL detection kit (Amersham, Freiburg, Germany). Primary antibodies used were affinity-purified rabbit polyclonal RAX anti-DMP1 antibody as previously reported [13], DO-1 anti-p53 (Calbiochem, Lucerne, Switzerland), or anti-β-actin antibodies (Sigma-Aldrich).

### 3.4. Luciferase Reporter Cloning and Assay

The 5′ flanking sequence of the human *DMTF1* gene (nucleotides −2350/+98) was PCR-amplified using the GC-RICH PCR System (Roche Diagnostics), genomic DNA from 293T cells as template and the following primers, *SacI/XhoI* restriction site sequences are underlined: Forward 5′-GGAGCTCTTCATTCCTCCATTAGCACAGCAATCTCCATCAGC-3′ and reverse 5′-GCTCGAGTCCGGGCACTTTGGAAGAACCAGGATGGAAGCTC-3′. Human *DMTF1* promoter deletion fragments were generated using the following primers: Forward 5′-GGAGCTCTTCACAGAGGACACATTTCATCAAG-3′ (−495/+98), forward 5′-GGAGCTCTCAAGTATGAAGACACACACTCCCTG-3′ (−418/+98), forward 5′-GGAGCTCTCAGCAGTGGGATAGTCAGTGCCGAG-3′ (−232/+98) and the reverse primer described above. PCR fragments were TOPO cloned and *SacI/XhoI* fragments were further subcloned into pGL2-basic Luciferase vector (Promega) using standard cloning techniques.

An hDMP1 promoter MCF7 reporter cell line (MCF7 DMP1_LUC) was generated as follows: The hDMP1 deletion mutant (−495/+98) together with the *Luciferase* gene was PCR amplified using the following primers, *BamHI* restriction site sequence is underlined: Forward 5′-GGATCCTTCACAGAGGACACATTTCATCAAG-3′ and reverse 5′-GGATCCTTACAATTTGGACTTTCCGCCCTTC-3′. BamHI sites were added to the primers for subcloning into the pCR-XL-CS-hygro lentiviral vector [38]. MCF7 cells were transduced overnight, and 2 days later polyclonal pools were selected for 10 days with 250 mg/mL hygromycin B.

### 3.5. Transient Transfection

H1299, 293T, U2OS, and Schneider SL2 cells were transfected with Lipofectamine 2000 according to the manufacturer’s protocol (Invitrogen, Carlsbad, CA, USA). Briefly, cells were seeded 24 h prior to transfection to reach ~70–90% confluence at the time of transfection. Plasmid DNA and Lipofectamine 2000 (Thermo Fisher Scientific, Basel, Switzerland) were separately diluted in Opti-MEM (serum-free) and incubated for 5 min at room temperature. The diluted DNA and Lipofectamine 2000 were then combined and incubated for 15–20 min to allow complex formation. DNA-lipid complexes were added dropwise to cells in complete growth medium. The medium was replaced with fresh complete medium after 4–6 h. Cells were analyzed 24–48 h post-transfection. For 24-well plates, 293T, H1299, and U2OS cells were transfected with 0.2 µg plasmid DNA and 1.5 µL Lipofectamine 2000 per well, using a final complex volume of 100 µL Opti-MEM. For 6-well plates, SL2 cells were transfected with 4.4 µg plasmid DNA and 5 µL Lipofectamine 2000 per well, using a final complex volume of 250 µL Opti-MEM. Reporter expression was analyzed using the Dual-Luciferase Reporter Assay System (Promega). *Firefly* luciferase activity of each sample was normalized to its *Renilla* luciferase activity, and the fold activation was obtained by setting the value of empty vector control as 1.0.

### 3.6. Chromatin Immunoprecipitation (ChIP)

ChIP assays were performed according to the EZ-ChIP protocol (Millipore, Hampshire, UK). Following DNA purification, PCR was performed using a Hot Start Polymerase system (Fermentas, Nunningen, Switzerland) and primers comprising the EGR/SP1 site in the hDMP1 promoter (forward: 5′-AGCCGGAAGTGACGCGTACA-3′; reverse: 5′-AGTGGCTGCAGCTGGAGTGAG-3′).

### 3.7. Electrophoretic Mobility Shift Assay (EMSA)

The p53 protein was synthesized in vitro using rabbit reticulocyte lysates (TnT Quick Coupled Transcription/Translation System, Promega). Annealed probes were radioactively labeled using 50 mCi of adenosine 5′-[g-^32^P]triphosphate at 6000 Ci/mmol (Amersham, Zurich, Switzerland) and T4-Polynucleotide Kinase (Invitrogen). The probes used were 2xp53CON (5′-AGCTTAGACATGCCTAGACATGCCTA-3′; positive control; [40]), and hDMP1.PRE A (5′-AATTAGTCAAACATGTCGATCCTCCAGTAAAGCAAAGCATTTGT-3′) and B (5′-CAGAGGACACATTTCATCAAGAACGACTTAACACGCCCAGATATCTCATATTATGCCCCTGC-3′). Binding reactions were carried out in a total reaction volume of 15 µL containing 100 ng poly-(dIdC), 1 µL hot probe, 5 µL of p53 programmed reticulocyte lysate, 200 ng of monoclonal anti-p53 antibody PAb421 (Calbiochem), and competing cold oligonucleotides where indicated, in binding buffer (10 mM TrisHCl (pH 8.0), 250 mM KCl, 500 mM EDTA, 0.1% Triton-X 100, 12.5% glycerol (*v*/*v*), 200 mM DTT) for 30 min at room temperature. Protein-DNA complexes were separated on 4% non-denaturing PAG for 90 min at 1 mA/cm at 4 °C. Gels were dried and exposed to Kodak BioMax XAR Films at −80 °C.

## Data Availability

The original contributions presented in this study are included in the article/Supplementary Material. Further inquiries can be directed to the corresponding authors.

## Supplementary Materials

The following supporting information can be downloaded at: https://www.mdpi.com/article/10.3390/ijms27031344/s1.
