# Supplementary material for: Identification of DMP1 as Novel p53 Repressed Transcriptional Target
Source: Int J Mol Sci. 2026 Jan 29;27(3):1344. doi: 10.3390/ijms27031344 (PMC12897804; doi:10.3390/ijms27031344)
Supplement: Supplementary file 1 [file ijms-27-01344-s001.zip › ijms-4070182-supplementary.pdf]

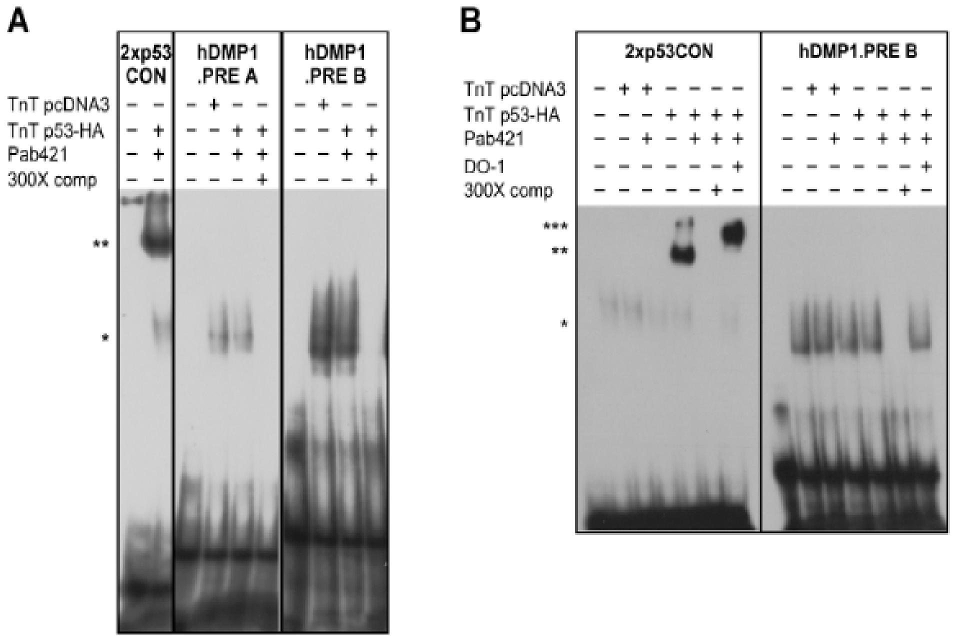

Supplementary Figure S1. p53 does not bind to two putative p53-binding sites in the hDMP1 promoter. Electrophoretic mobility shift assay (EMSA) using *in vitro* translated p53 protein and radiolabeled DNA probes. (**A**) Binding of p53 to a consensus p53-binding site (2×p53CON, positive control) and to two putative hDMP1 promoter elements (PRE A and PRE B). (**B**) EMSA performed with 2×p53CON and hDMP1 PRE B in the presence of anti-p53 antibody DO-1 to assess supershift formation. *, nonspecific bands; **, p53–DNA complex; ***, p53 supershift.
